# A spontaneous gravity prior: newborn chicks prefer stimuli that move against gravity

**DOI:** 10.1098/rsbl.2022.0502

**Published:** 2023-02-08

**Authors:** Larry Bliss, Vera Vasas, Laura Freeland, Robyn Roach, Elisa Raffaella Ferrè, Elisabetta Versace

**Affiliations:** ^1^ Department of Biological and Experimental Psychology, Queen Mary University of London, London E1 4NS, UK; ^2^ Department of Psychological Sciences, Birkbeck, University of London, London WC1E 7HX, UK; ^3^ Alan Turing Institute, London NW1 2DB, UK

**Keywords:** predispositions, upward movement, precocial species, chicks, gravity, animacy

## Abstract

At the beginning of life, inexperienced animals use evolutionary-given preferences (predispositions) to decide what stimuli to attend and approach. Stimuli that contain cues of animacy, such as face-like stimuli, biological motion and changes in speed, are particularly attractive across vertebrate taxa. A strong cue of animacy is upward movement against terrestrial gravity, because only animate objects consistently move upward. To test whether upward movement is spontaneously considered attractive already at birth, we tested the early preferences of dark-hatched chicks (*Gallus gallus*) for upward- versus downward-moving visual stimuli. We found that, without any previous visual experience, chicks consistently exhibited a preference to approach stimuli that move upward, against gravity. A control experiment showed that these preferences are not driven by avoidance of downward stimuli. These results show that newborn animals have a gravity prior that attracts them toward upward movement. Movement against gravity can be used as a cue of animacy to orient early approach responses in the absence of previous visual experience.

## 1. Background

From the earliest stages of life, the ability to detect living beings is crucial for survival: living beings can give support (e.g. a mother hen provides warmth, protection and resources to chicks) or pose a threat (e.g. an approaching predator). In fact, early predispositions to approach/attend to cues of animacy have been found in young and inexperienced infants and other animals at the beginning of life [1–3]. These predispositions work as priors that help young animals to direct their attention towards animate objects instead of inanimate distractors. For instance, soon after birth, infants, chicks and tortoises preferentially attend to face-like stimuli [4–7], infants and chicks prefer to approach objects that change their speed [8], move along the longer body-plan axis [9,10] (but see [11] for pecking preferences) or move in accordance with biological motion [12,13], as animate objects do. Young chicks align their body with the right–left direction of biological motion (point-light displays that move in a semi-rigid relationship across multiple joints of an animal) of a moving animal only when its vertical axis is upright [14]. The presence of predispositions that support the detection of animate objects raises the question whether the presence of upward movement against terrestrial gravity (irrespective of biological motion) is used as an indicator of animacy. In fact, only animate objects can consistently move upward, against gravity, on their own [15]. Accordingly, human adults find that upward-moving stimuli appear more animate than downward-moving stimuli [16]. However, it is not known whether the association of upward movement and animacy is shared with non-human animals. It remains an open question whether animals require experience to associate upward trajectories with animate objects, or whether upward movements are attractive from birth, as an evolutionary-given preference.

**Figure 1.**
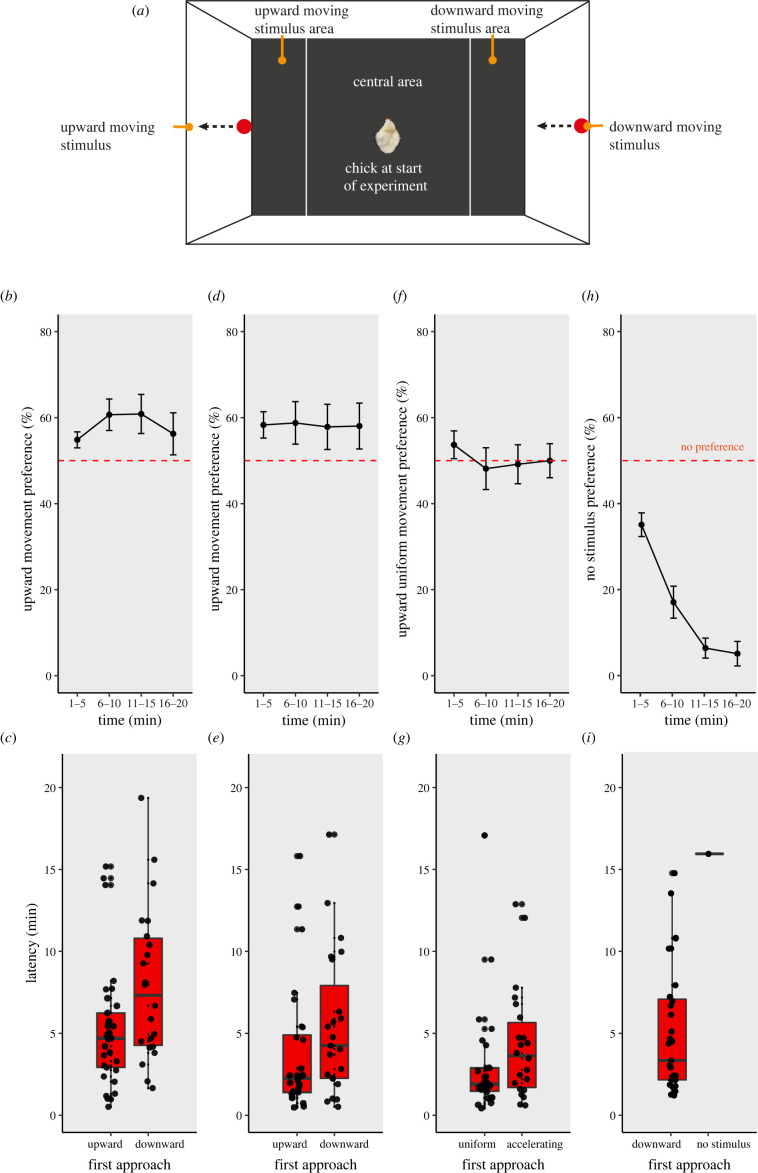
Chicks prefer upward-moving stimuli. (*a*) Apparatus. The arena was divided into two stimulus areas and a central area. The position of the chick was recorded. (*b*) Preference in each 5 min time bin and (*c*) latency for upward versus downward moving, accelerating stimuli in Exp. 1. (*d*) Preference and (*e*) latency for upward versus downward constant speed movement in Exp. 2. (*f*) Preference and (*g*) latency for upward uniform versus upward accelerating movement in Exp. 3. (*h*) Preference and (i) latency for downward accelerating stimulus versus no stimulus in Exp. 4. Bar plots and line plots show mean +/- s.e.m. of preference. Box plots display median, quartiles and outliers.

To clarify whether newborn animals can use the upward direction of movement as a cue of animacy, we investigated whether newly hatched, visually inexperienced chicks (*Gallus gallus*) are spontaneously attracted by movement against gravity. Chicks are born with a mature sensory and motor system and have a strong motivation to approach social partners [1]. Soon after hatching in complete darkness, with no experience with moving objects, we tested neonates' preference to approach an upward-moving versus a downward-moving stimulus (a red circle attractive for chicks), presented on two opposite monitors for 20 min, divided in four consecutive time bins (figure 1*a*). Using automated tracking [17], we analysed the percentage of time spent close to the stimulus moving against gravity in each time bin, and the first-approach latency (time before entering a stimulus area).

## 2. Methods

At the end of incubation, chicks (*Gallus gallus* of both sexes, 55 in Exp. 1, 51 in Exp. 2, 48 in Exp. 3 and 31 in Exp. 4) hatched in darkness in individual compartments. Each chick was tested once for 20 min (four consecutive 5 min bins) in a rectangular arena virtually divided in a starting area (54 × 60 cm), where the chick was initially located, and two stimulus areas (18 × 60 cm) (figure 1*a*). In the stimulus areas, two different video stimuli were simultaneously played in a loop on computer monitors (Asus MG248, 120 Hz), with left/right side counterbalanced between subjects. In Exp. 1, a red circle (Ø 3.28 cm) moved vertically upward or downward along the centre, with 9.81 m s^−2^ acceleration (electronic supplementary material, movie S1). In Exp. 2, the same stimuli moved at uniform speed (3.57 m s^−1^) (electronic supplementary material, movie S2). Exp. 3 used the accelerating upward stimulus from Exp. 1 and the uniform upward stimulus from Exp 2 (electronic supplementary material, movie S3). In Exp. 4, one stimulus moved downwards at the acceleration of terrestrial gravity on one screen, with the other screen blank (electronic supplementary material, movie S4). Chicks’ centroid position in the arena was automatically tracked with DeepLabCut [17] and used to identify the position of the chick. Individual chicks' preference was calculated as a percentage, where 100 indicated a complete preference for the largest stimulus, 0 for the alternative stimulus and 50 no preference. Formore detail see electronic supplementarymaterial, S1 Extended Methods.

Friedman tests showed no significant difference across the four 5 min time bins in Exp. 1–3 (Exp. 1, *Q* = 4.40, *p* = 0.221; Exp. 2: *Q* = 0.34, *p* = 0.952; Exp. 3: *Q* = 2.67, *p* = 0.446). Thus, in Exp. 1–3, we calculated an overall preference index for each chick as an average of the four bins and tested the preference against chance level with a one-sample Wilcoxon test. The latency to first approach (time elapsed between the start of the test and the first stimulus area entered by each chick) was assessed using a Mann–Whitney *U*-test. In Exp. 4, the Friedman test detected a significant difference across time bins (*Q* = 56.56, *p* < 0.001), so the time bins were assessed individually with a Wilcoxon test. In Exp. 4, only one chick chose the no stimulus option, and for this reason differences in latencies could not be analysed. All tests were two-tailed; significance was set at *p* ≤ 0.05. Analyses were run in Python, figures were prepared using R.

## 3. Results

### (a) Chicks spontaneously approach upward-moving stimuli

In Exp. 1, we tested the preference for upward versus downward movement, presenting chicks with red circles that moved according to terrestrial gravity acceleration (9.81 m s^−2^) in opposite vertical directions (electronic supplementary material, movies S1 and S5, see [18]). Chicks significantly preferred the upward-moving stimulus: median 60.5%, *W* = 476, *p* = 0.014 (figure 1*b*). The first-approach latency was shorter for chicks that moved towards the upward stimulus (4.70 min versus 7.33 min, *U* = 230, *p* = 0.011, figure 1*c*). These results show that upward-moving stimuli are more attractive than downward-moving stimuli. In Exp. 2, we tested the preference for upward versus downward uniform (constant speed) movement (electronic supplementary material, movie S2). Chicks preferred the upward-moving stimuli (median: 63.2%, *W* = 448, *p* = 0.044, figure 1*d*), showing that the mere upward direction of movement (without acceleration) is sufficient to elicit a spontaneous preference. Again, chicks had a shorter first-approach latency for upward-moving stimuli (median: 2.25 versus 4.25 min; *U* = 234, *p* = 0.049, figure 1*e*). In Exp. 3, we directly compared the responses to accelerating versus uniformly moving upward stimuli of the same average speed (electronic supplementary material, movie S3). Chicks showed no preference, spending 48.5% of time (median) at the uniform moving stimulus (*W* = 587, *p* = 0.992, figure 1*f*). The first-approach latency was faster for the uniform upward movement (median: 1.90 versus 3.63 min; *U* = 206, *p* = 0.050, figure 1*g*), suggesting that chicks might be attracted to the faster movement of the uniform stimulus at ground level (electronic supplementary material, movie S3). Overall, our results show that upward accelerating and constant speed movements are attractive for visually inexperienced chicks.

### (b) Chicks do not avoid downward stimuli

Chicks are predisposed to flee away from fast-looming approaching stimuli [19]. To test whether the preference that we observed for upward-moving stimuli was a fleeing response to downward, approaching moving stimuli, in Exp. 4, we tested chicks’ preference for downward accelerating movement versus a blank screen with no stimuli (electronic supplementary material, movies S4 and S6). Chicks exhibited a very strong preference for the downward stimulus (median at 1–5 min = 38.8, median at 6–10 min = 1.7, *p* < 0.001; median at subsequent times = 0, *p* < 0.001, figure 1*h,i*). These results show that the downward-moving stimulus was not aversive.

## 4. Discussion

The ability to discriminate between animate and inanimate objects is adaptive from early life, enabling animals to identify relevant information, find support from conspecifics and avoid threats [1,15]. Upward movement against gravity is a clear—yet neglected—cue of animacy, since in most situations inanimate objects either remain still or move along gravity (e.g. a falling rock). We found that young, visually inexperienced chicks are attracted by stimuli that move upward, against gravity. These findings indicate that chicks have a spontaneous, non-learned predisposition for upward-moving stimuli, suggesting that upward-moving stimuli are used as a cue of animacy. Control experiments showed that the preference for upward-moving stimuli does not depend on the presence of acceleration. In fact, chicks prefer upward versus downward stimuli irrespective of acceleration patterns and respond even faster to uniform movement. Moreover, the preference for upward movement is not driven by aversion to downward stimuli, since chicks were attracted by downward-moving stimuli, when the other option was the absence of stimuli. Crucially, as chicks were completely visually naive, our results show that the preference for upward movement is present in the absence of previous visual experience. A gravity prior operates from the beginning of life, before any experience with moving objects [3]. These results pave the way to further studies on whether other species, including humans, are spontaneously attracted by upwards movement from early stages of life and on what dynamics and trajectories incompatible with passive movement along terrestrial gravity are attractive.

## Data Availability

Data and scripts are included in the article and electronic supplementary material. All raw data and analysis scripts are available at 10.5281/zenodo.7437294 [20]. Data were collected with the methods described in the manuscript. Raw values are stored in .csv files. Analysis was conducted using Python scripts. Data files and analysis scripts are described individually in read-me files at 10.5281/zenodo.7437294. The data are provided in the electronic supplementary material [21].
